# Supplementary material for: GPCRome-wide analysis of G-protein-coupling diversity using a computational biology approach
Source: Nat Commun. 2023 Jul 19;14:4361. doi: 10.1038/s41467-023-40045-y (PMC10356834; doi:10.1038/s41467-023-40045-y)
Supplement: Supplementary file 1 — Supplementary Information [file 41467_2023_40045_MOESM1_ESM.pdf]

GPCRomE-wide analysis of G-protein-coupling diversity using a computational biology approach

Marin Matic[1]*, Pasquale Miglionico[1]*, Manae Tatsumi[2]*, Asuka Inoue[2], Francesco Raimondi[1]

1 Laboratorio di Biologia Bio@SNS, Scuola Normale Superiore

2 Graduate School of Pharmaceutical Sciences, Tohoku University, Sendai, Miyagi 980-8578, Japan

Supplementary Information

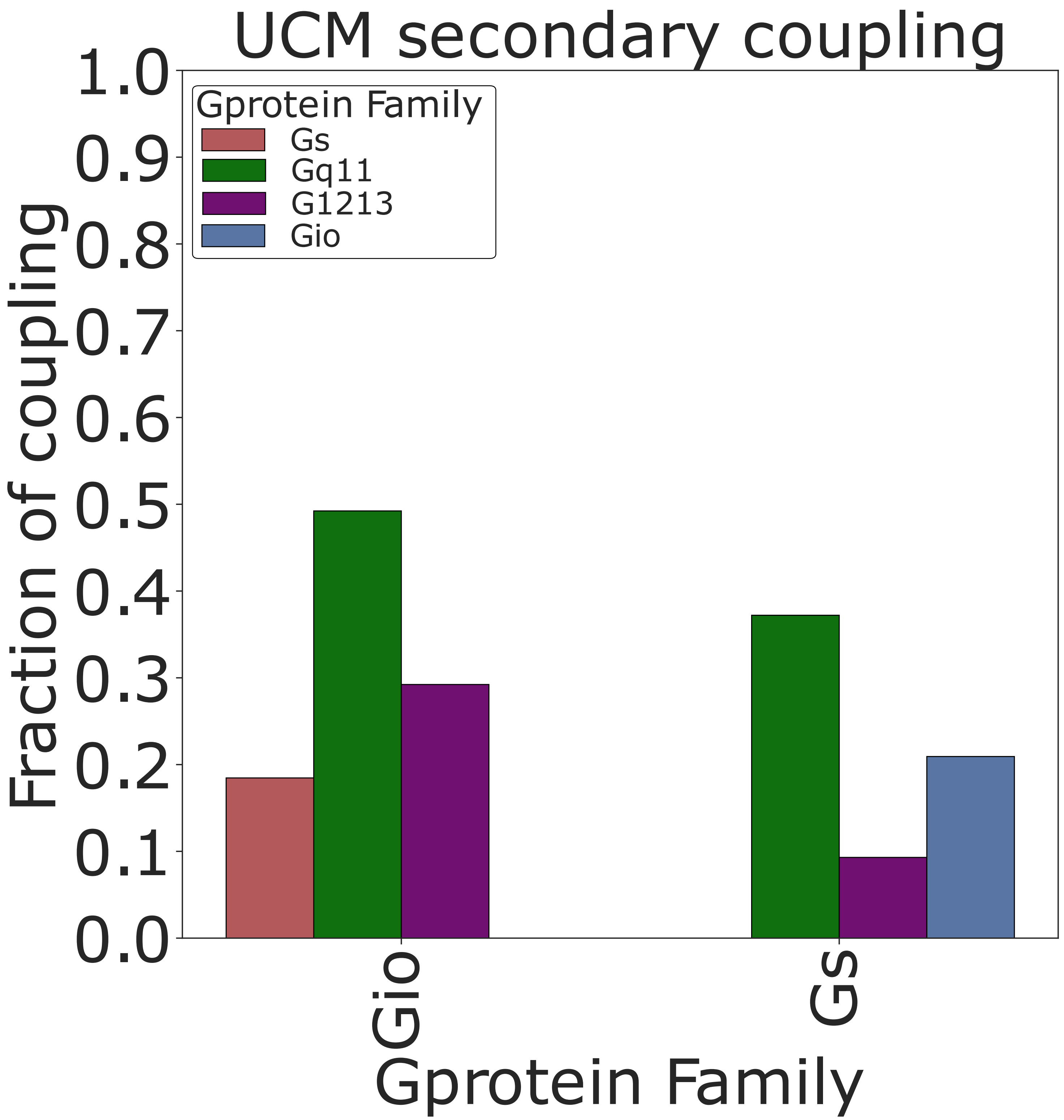

Supplementary Figure1: secondary couplings from UCM of Gs (left) and Gi/o coupled receptors. Bars indicate the value as a fraction over the total number of receptors in the UCM dataset.

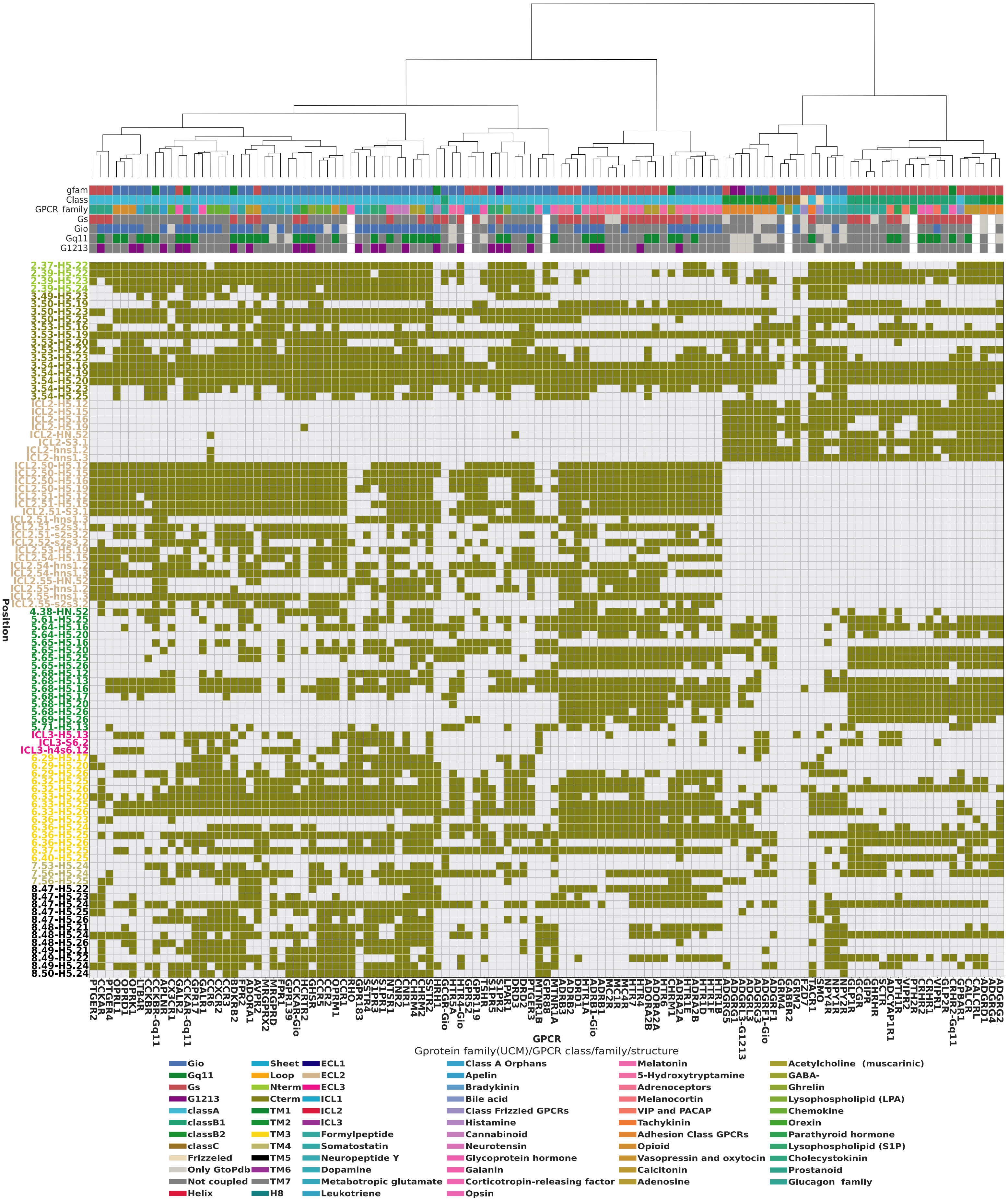

Supplementary Figure2: Full GPCR-G-protein contact interface fingerprint (or CF fingerprint): each row is a GPCR-G-protein contact positions (referenced respectively to GPCRdb numbering and G-protein position (CGN) numbering) and each column is a unique receptor. If a receptor is complexed with more than one G-protein, its complex fingerprint is reported accordingly. Columns are color annotated to indicate: G-protein bound in the experimental structure, GPCR class, experimental reported coupling (according to UCM, or either GEMTA, Shedding or GtoPDB). Only contacts present in at least 20% of the structures are shown, considering all unique complexes with the four G-protein families. Source data are provided as a Source Data file.

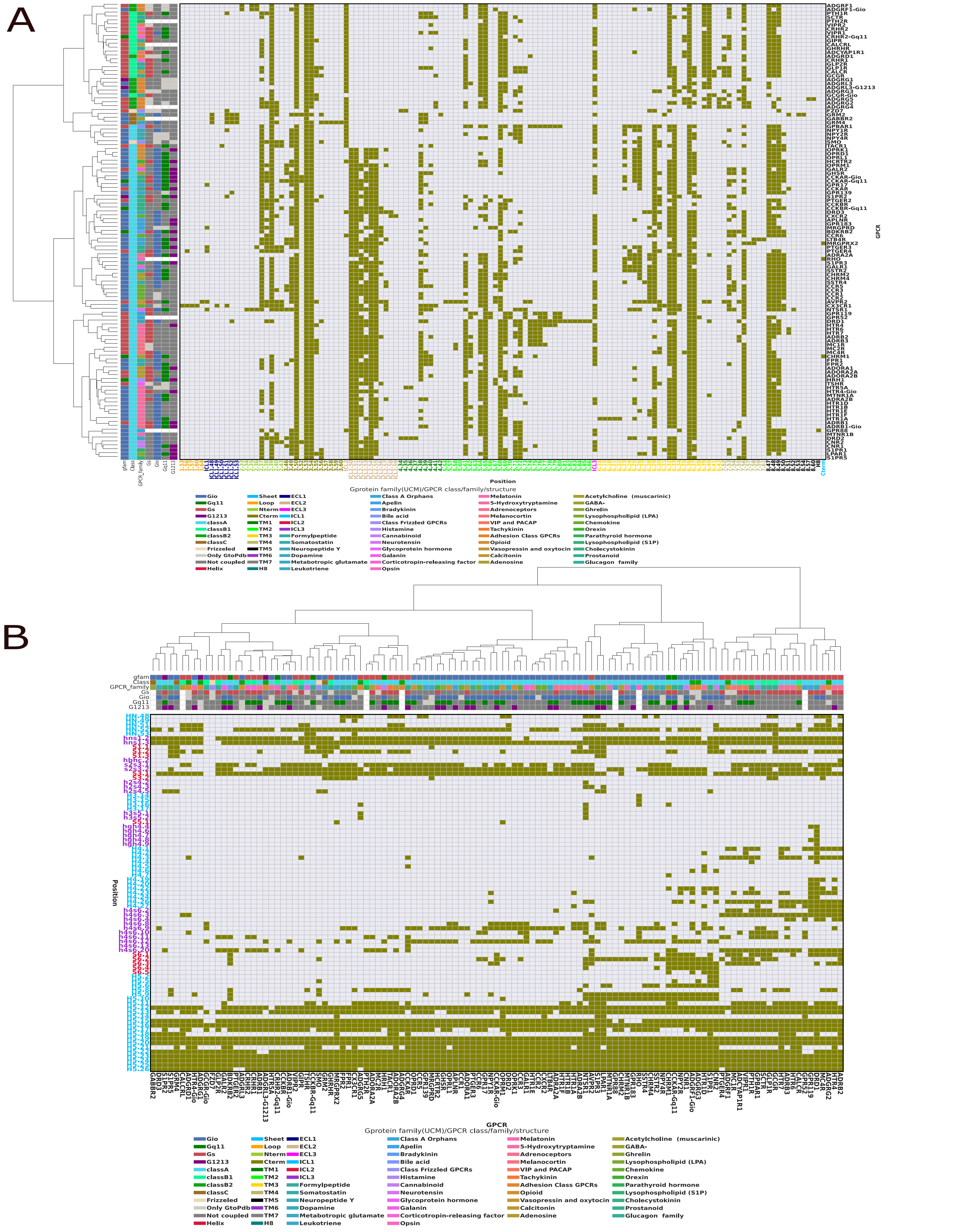

Supplementary Figure3: Fingerprint clustering for A) receptor and B) G-protein contacting position. Each column is a GPCR (or G-protein) contact position (referenced respectively to GPCRdb and G-protein position (CGN) numbering) and each row is a unique receptor. If a receptor is complexed with more than one G-protein, its complex fingerprint is reported accordingly. Rows are color annotated as Supplementary Figure2's columns. Source data are provided as a Source Data file.

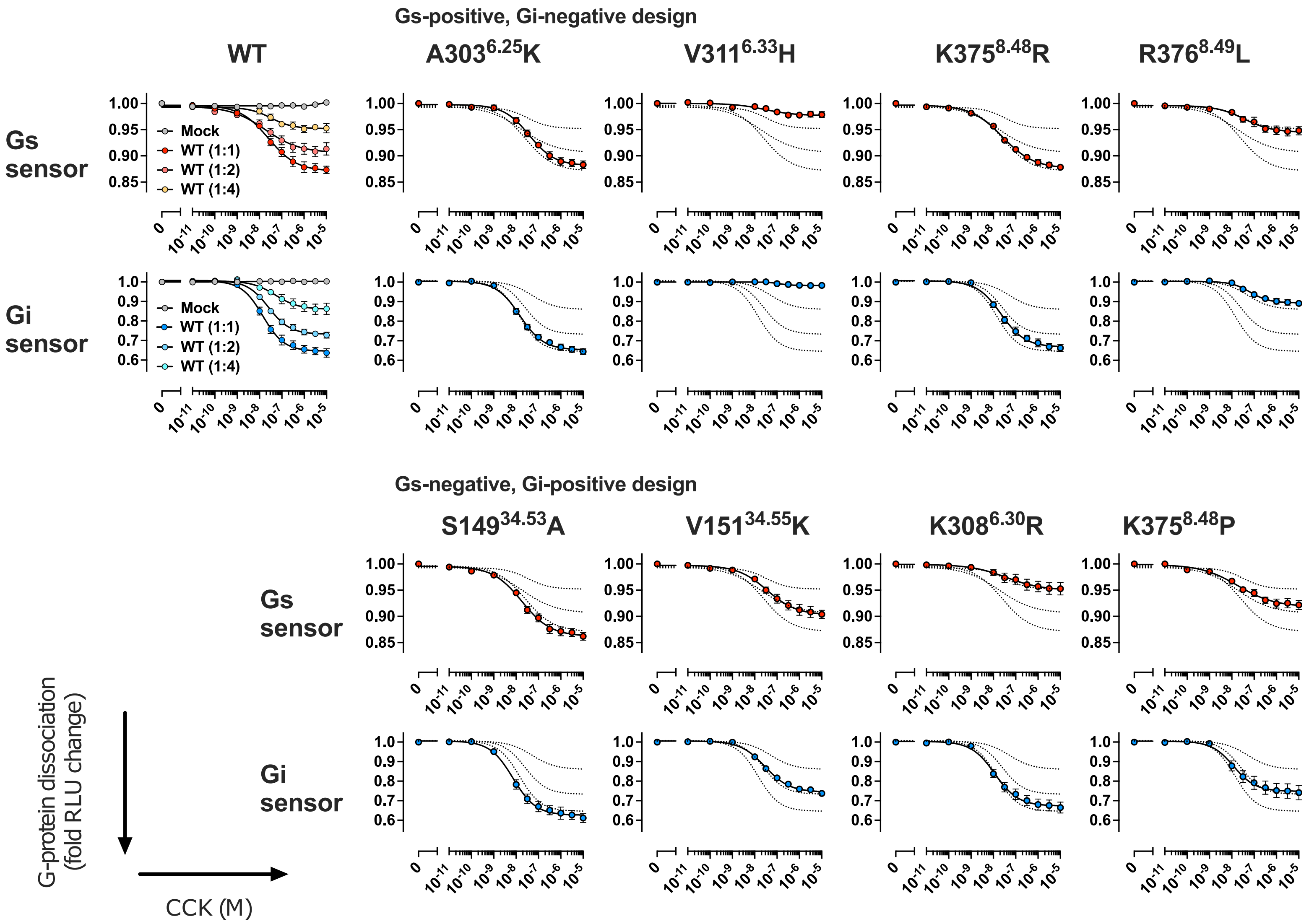

Supplementary Figure4: Concentration-response curves of the CCKAR mutants for Gs and Gi1 activation. HEK293 cells transiently expressing the indicated CCKAR construct along with the NanoBiT-Gs or the NanoBiT-Gi1 sensor were subjected to the NanoBiT-G-protein-dissociation assay using CCK-8 as a ligand. WT (1:2) and WT (1:4) denote 2-fold and 4-fold less volumes, respectively, of transfected plasmids than the mutant plasmids. Data are from 3-5 independent experiments with error bars representing SEM. Dotted lines in the mutant panels denote sigmoidal responses of WT (1:1), WT (1:2) and WT (1:4).

Supplementary Figure5: RMSD hierarchical clustering for all the 362 structures analysed. Color annotations are as in Figure 4A. Source data are provided as a Source Data file.

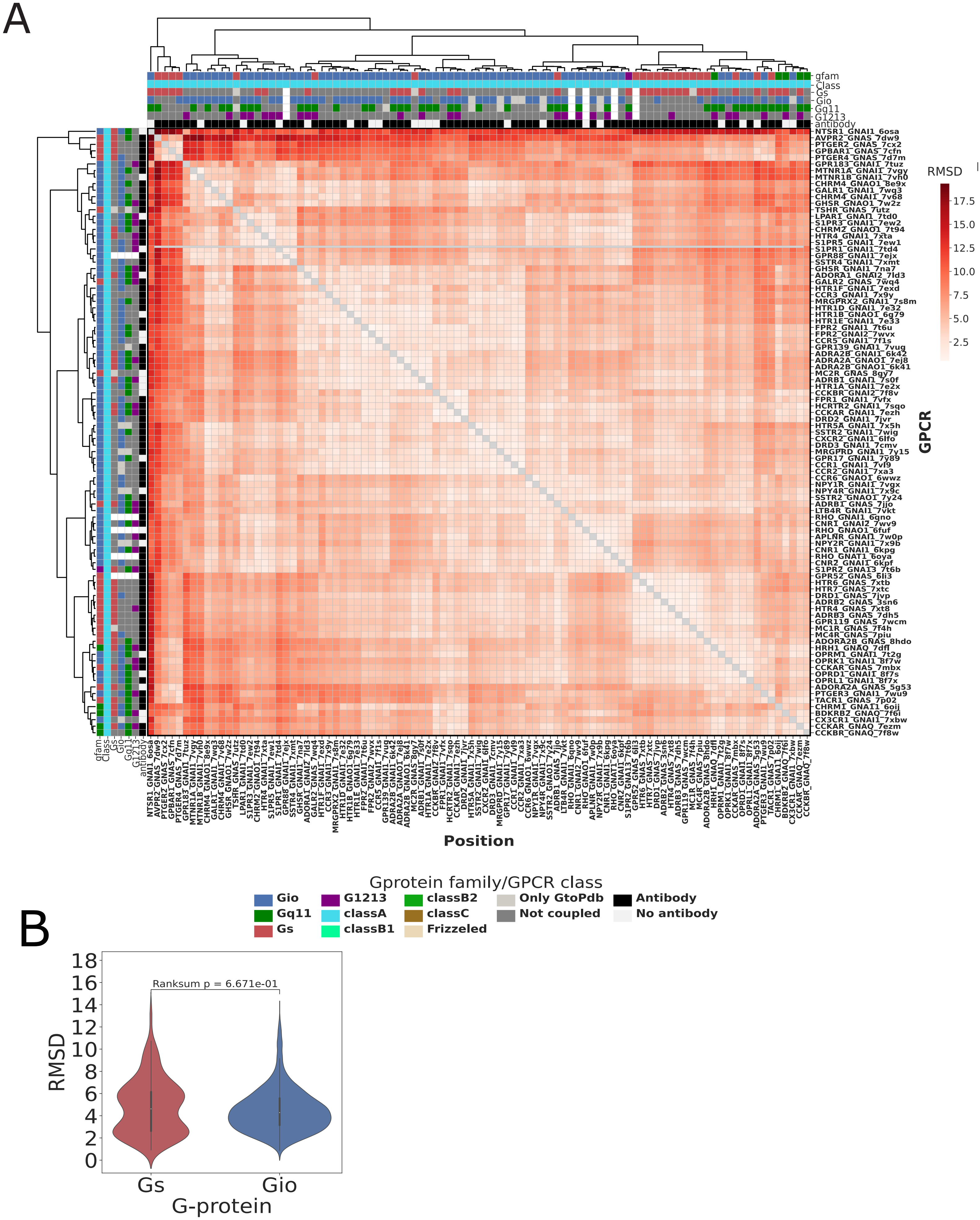

Supplementary Figure6: RMSD analysis of unique complexes of Class A receptors. A) RMSD hierarchical clustering; B) RMSD distributions of Gs and Gi/o complexes; n=2080 experimental structure pairs for Gi/o and n=203 experimental structure pairs for Gs; statistics is performed via a Two-sided Wilcoxon rank-sum test with Bonferroni correction (P=6.671E-1). Boxplots show the median as the centre and first and third quartiles as bounds of the box; the whiskers extend to the last data point within 1.5 times the interquartile range (IQR) from the box's boundaries. Source data are provided as a Source Data file.

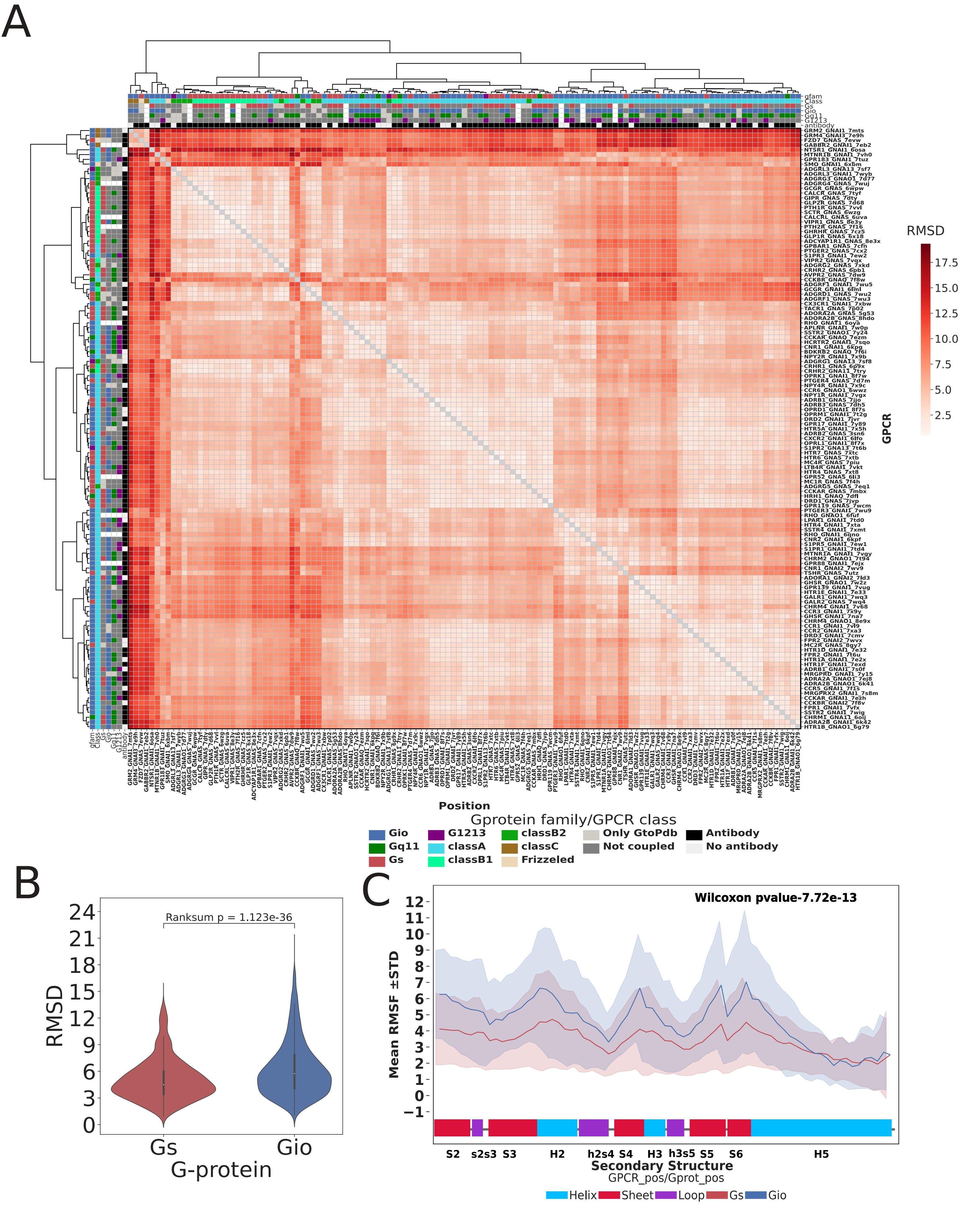

Supplementary Figure7: RMSD analysis of representative structures of unique complexes after Rosetta relaxation. A) RMSD hierarchical clustering; B) RMSD distributions of Gs and Gi/o complexes; n=2628 experimental structure pairs for Gi/o and n=903 experimental structure pairs for Gs, statistics is performed via a Two-sided Wilcoxon rank-sum test with Bonferroni correction (P=1.123E-36).Boxplots show the median as the centre and first and third quartiles as bounds of the box; the whiskers extend to the last data point within 1.5 times the interquartile range (IQR) from the box's boundaries. C) Root mean squared fluctuations of the G-protein consensus positions, each point is represented as mean±SD and each point has n=125 experimental structure to calculate mean, statistics is performed via a two-sided Wilcoxon rank-sum test with Bonferroni correction(P=7.72E-13). Source data are provided as a Source Data file.

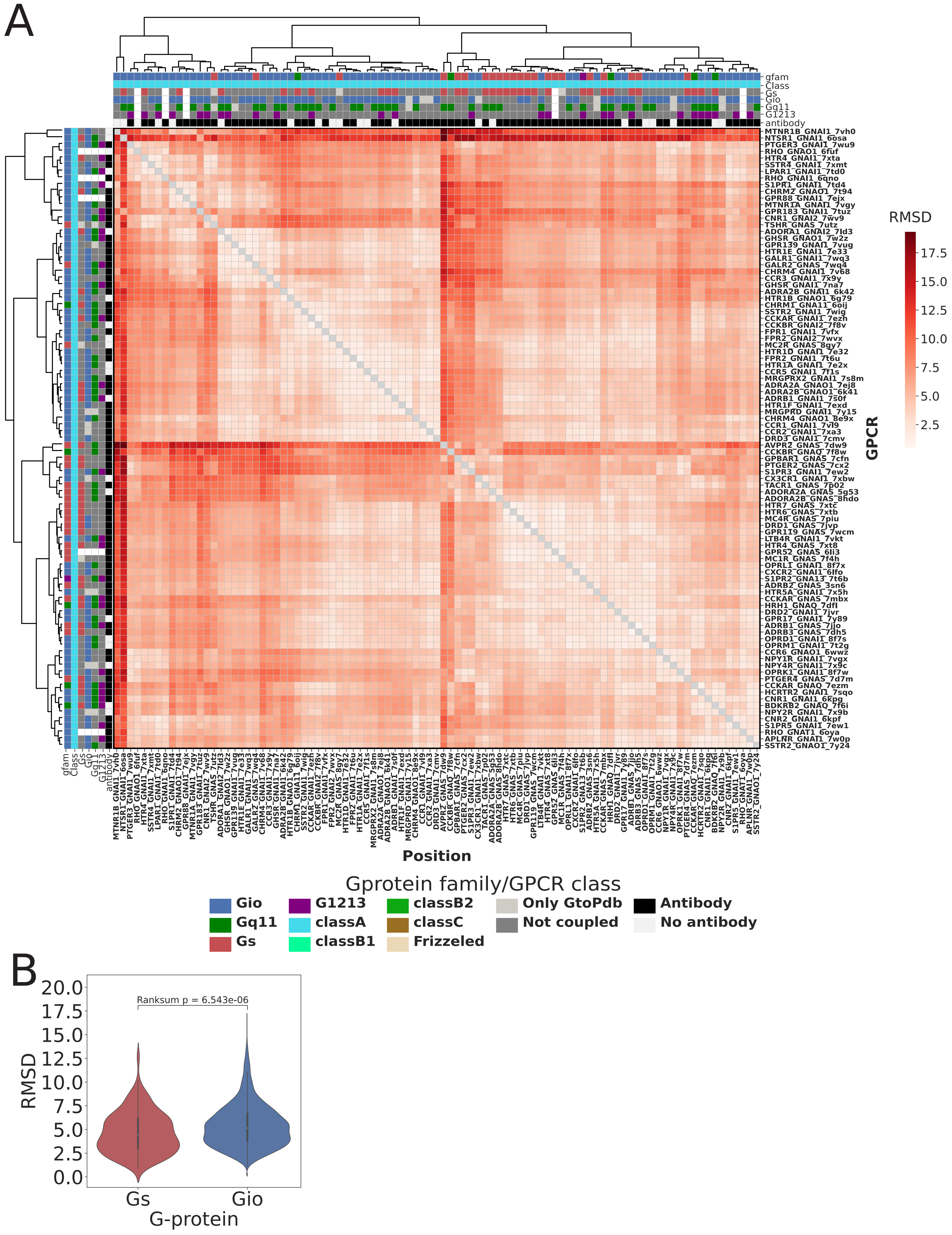

Supplementary Figure8: RMSD analysis of representative structures of unique complexes of Class A receptors after Rosetta relaxation. A) RMSD hierarchical clustering; B) RMSD distributions of Gs and Gi/o complexes, n=2080 experimental structure pairs for Gio and n=203 experimental structure pairs for Gs, statistics is performed via a Two-sided Wilcoxon rank-sum test with Bonferroni correction (P=6.543E-6). Boxplots show the median as the centre and first and third quartiles as bounds of the box; the whiskers extend to the last data point within 1.5 times the interquartile range (IQR) from the box's boundaries. Source data are provided as a Source Data file.

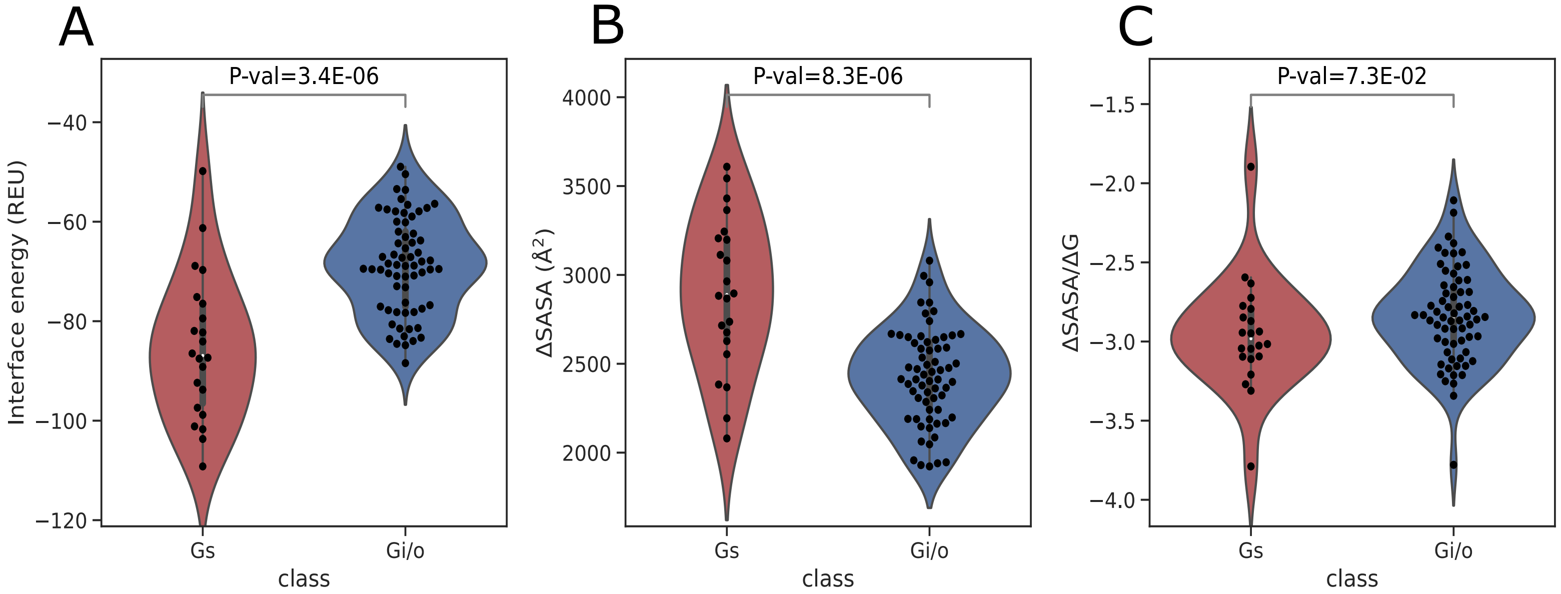

Supplementary Figure9: Binding energy estimated through Rosetta for class A receptor bound to Gs and Gi/o proteins A) ΔG binding (RU); B) Delta Solvent Accessible Surface Area (ΔSASA); C) ΔSASA /ΔG; .n=22 complexes for Gs and n=65 complexes for Gi/o. The p-values have been computed with a two-sided Mann–Whitney U test. Boxplots show the median as the centre and first and third quartiles as bounds of the box; the whiskers extend to the last data point within 1.5 times the interquartile range (IQR) from the box's boundaries.
