# Supplementary material for: GPCRome-wide analysis of G-protein-coupling diversity using a computational biology approach
Source: Nat Commun. 2023 Jul 19;14:4361. doi: 10.1038/s41467-023-40045-y (PMC10356834; doi:10.1038/s41467-023-40045-y)
Supplement: Supplementary file 3 — Description of Additional Supplementary Files [file 41467_2023_40045_MOESM3_ESM.pdf]

**File name: Supplementary Data 1**

Description: list of PDB structures considered for the study

**File name : Supplementary Data 2**

Description: chains corresponding to G-protein from representative PDB and residue number mapping to Common G-protein numbering

**File name: Supplementary Data 3**

Description: chains corresponding to GPCRs from representative PDB and residue number mapping to GPCRdb numbering

**File name: Supplementary Data 4**

Description: Contact interfaces of CCKAR structures(7EZH and 7EZK) used in Multi-State design. Mutated sequences for $G_s$ and $G_{i/o}$ negative design and binding energy of the mutated structures (InterfaceAnalyzer-Rosetta)

**File name: Supplementary Data 5**

Description: binary GPCR-Gα subunit complexes predicted through AlphaFold-multimer with Rosetta binding energy (InterfaceAnalyzer) and structural filter annotations
