# Supplementary material for: GPCRome-wide analysis of G-protein-coupling diversity using a computational biology approach
Source: Nat Commun. 2023 Jul 19;14:4361. doi: 10.1038/s41467-023-40045-y (PMC10356834; doi:10.1038/s41467-023-40045-y)
Supplement: Supplementary file 5 — Reporting Summary [file 41467_2023_40045_MOESM5_ESM.pdf]

Corresponding author(s): Francesco Raimondi
Last updated by author(s): Jul 4, 2023

Reporting Summary

Nature Portfolio wishes to improve the reproducibility of the work that we publish. This form provides structure for consistency and transparency in reporting. For further information on Nature Portfolio policies, see our Editorial Policies and the Editorial Policy Checklist.

Statistics

For all statistical analyses, confirm that the following items are present in the figure legend, table legend, main text, or Methods section.

n/a	Confirmed	
☐	☒	The exact sample size (*n*) for each experimental group/condition, given as a discrete number and unit of measurement
☐	☒	A statement on whether measurements were taken from distinct samples or whether the same sample was measured repeatedly
☐	☒	The statistical test(s) used AND whether they are one- or two-sided<br>*Only common tests should be described solely by name; describe more complex techniques in the Methods section.*
☐	☒	A description of all covariates tested
☐	☒	A description of any assumptions or corrections, such as tests of normality and adjustment for multiple comparisons
☐	☒	A full description of the statistical parameters including central tendency (e.g. means) or other basic estimates (e.g. regression coefficient) AND variation (e.g. standard deviation) or associated estimates of uncertainty (e.g. confidence intervals)
☐	☒	For null hypothesis testing, the test statistic (e.g. *F*, *t*, *r*) with confidence intervals, effect sizes, degrees of freedom and *P* value noted<br>*Give P values as exact values whenever suitable.*
☒	☐	For Bayesian analysis, information on the choice of priors and Markov chain Monte Carlo settings
☒	☐	For hierarchical and complex designs, identification of the appropriate level for tests and full reporting of outcomes
☒	☐	Estimates of effect sizes (e.g. Cohen's *d*, Pearson's *r*), indicating how they were calculated

Our web collection on statistics for biologists contains articles on many of the points above.

Software and code

Policy information about availability of computer code

Data collection	PDB<br>AlphaFold-multimer (v2.3.13732)<br>https://github.com/raimondilab/GPCR_structure_analysis
Data analysis	We employed Pymol (v2.4.1) and ChimeraX (v1.5) to generate 3D cartoon representations. We employed customize scripts in python (version 3.8.11), using matplotlib (v3.6.0), seaborn (v0.11.1) and biopython (v1.78) libraries. We calculated residuE-residue contact by using a customized script derived from the CIFPARSE-OBJ C++ library (https://mmcif.wwpdb.org/docs/sw-examples/cpp/html/index.html).

For manuscripts utilizing custom algorithms or software that are central to the research but not yet described in published literature, software must be made available to editors and reviewers. We strongly encourage code deposition in a community repository (e.g. GitHub). See the Nature Portfolio guidelines for submitting code & software for further information.

Data

Policy information about availability of data

All manuscripts must include a data availability statement. This statement should provide the following information, where applicable:

- Accession codes, unique identifiers, or web links for publicly available datasets
- A description of any restrictions on data availability
- For clinical datasets or third party data, please ensure that the statement adheres to our policy

Source data are provided with this paper.
The raw data used in this study are available in the Zenodo database under accession code https://doi.org/10.5281/zenodo.8063796.
Data generated for this study are also available at:
https://github.com/raimondilab/GPCR_structure_analysis (DOI: 10.5281/zenodo.8067369)
GPCR-Heterotrimeric G protein complexes predicted with AF-multimer are also available via Precogx webserver (https://precogx.bioinfolab.sns.it/).

Research involving human participants, their data, or biological material

Policy information about studies with human participants or human data. See also policy information about sex, gender (identity/presentation), and sexual orientation and race, ethnicity and racism.

Reporting on sex and gender	n/a
Reporting on race, ethnicity, or other socially relevant groupings	n/a
Population characteristics	n/a
Recruitment	n/a
Ethics oversight	n/a

Note that full information on the approval of the study protocol must also be provided in the manuscript.

Field-specific reporting

Please select the one below that is the best fit for your research. If you are not sure, read the appropriate sections before making your selection.

☒ Life sciences ☐ Behavioural & social sciences ☐ Ecological, evolutionary & environmental sciences

For a reference copy of the document with all sections, see nature.com/documents/nr-reporting-summary-flat.pdf

Life sciences study design

All studies must disclose on these points even when the disclosure is negative.

Sample size	Regarding the structural bioinformatic analysis, we considered a set of 125 non-redundant structures, starting from a total of 362. We also considered 825 high quality predicted structures, from a total of 996 predicted GPCR-G protein binary interactions. For the experimental validation, 5 independent experiments were performed, with the exception of WT (1:4), for which 3 independent experiments were performed
Data exclusions	We excluded from the analysis either experimental structures with low resolution or involving a chimeric receptor, or structures predicted with low quality,as detailed in the manuscript
Replication	For the experimental validation, 5 independent experiments were performed, with the exception of WT (1:4), for which 3 independent experiments were performed
Randomization	No randomization is typically required for the structural bioinformatic analysis and in-vitro assays used for this study
Blinding	No blinding is typically required for the structural bioinformatic analysis and in-vitro assays used for this study

Reporting for specific materials, systems and methods

We require information from authors about some types of materials, experimental systems and methods used in many studies. Here, indicate whether each material, system or method listed is relevant to your study. If you are not sure if a list item applies to your research, read the appropriate section before selecting a response.

Materials & experimental systems

n/a	Involved in the study
☐	☒ Antibodies
☐	☒ Eukaryotic cell lines
☒	☐ Palaeontology and archaeology
☒	☐ Animals and other organisms
☒	☐ Clinical data
☒	☐ Dual use research of concern
☒	☐ Plants

Methods

n/a	Involved in the study
☒	☐ ChIP-seq
☒	☐ Flow cytometry
☒	☐ MRI-based neuroimaging

Antibodies

Antibodies used

anti-FLAG epitope (DYKDDDDK) tag monoclonal antibody (Clone 1E6, FujiFilm Wako Pure Chemicals, cat no. 012-22384; working dilution at 10 µg per ml); anti-mouse IgG secondary antibody conjugated with Alexa Fluor 488 (Thermo Fisher Scientific, cat no. A11001; working dilution at 10 µg per ml).

Validation

Describe the validation of each primary antibody for the species and application, noting any validation statements on the manufacturer's website, relevant citations, antibody profiles in online databases, or data provided in the manuscript.

Eukaryotic cell lines

Policy information about cell lines and Sex and Gender in Research

Cell line source(s)

HEK293A cells (Thermo Fisher Scientific, cat no. R70507)

Authentication

Describe the authentication procedures for each cell line used OR declare that none of the cell lines used were authenticated.

Mycoplasma contamination

Confirm that all cell lines tested negative for mycoplasma contamination OR describe the results of the testing for mycoplasma contamination OR declare that the cell lines were not tested for mycoplasma contamination.

Commonly misidentified lines
(See ICLAC register)

Name any commonly misidentified cell lines used in the study and provide a rationale for their use.
